# An Exploratory Trial of Brief Mindfulness-Based Zentangle Art Workshops in Family Social Services during COVID-19: Transitioning from Offline to Online

**DOI:** 10.3390/ijerph191710926

**Published:** 2022-09-01

**Authors:** Shirley Man-Man Sit, Ellen Ng, Hilary Pui-Yee Ho, Peony Cheuk-Yeuk Wong, Man-Ping Wang, Sai-Yin Ho, Tai-Hing Lam, Agnes Yuen-Kwan Lai

**Affiliations:** 1 School of Public Health, The University of Hong Kong, Hong Kong, China; 2 School of Nursing, The University of Hong Kong, Hong Kong, China; 3 Caritas-Hong Kong, Hong Kong, China

**Keywords:** Zentangle, art therapy, mindfulness, family social services, information and communication technology, community-based, COVID-19

## Abstract

Mindfulness-based art therapy has shown to improve psychological well-being. Zentangle is an easy-to-learn, mindfulness-based art therapy suitable for everyone. We reported the transition from face-to-face to online Zentangle workshops in family social services during COVID-19. We explored feedback from face-to-face workshops and the acceptability of an online approach utilizing information communication technology (ICT) to achieve greater service reach, satisfaction, and knowledge and related outcomes. Under the Hong Kong Jockey Club SMART Family-Link Project and in collaboration with Caritas Integrated Family Service Centre—Aberdeen, this study was conducted in two phases: a four-session, face-to-face workshop (phase one) and eleven online single-session workshops (phase two) from September 2019 to September 2020. A total of 305 participants joined the workshops. Phase one participants (*n* = 11) reported high satisfaction (4.7 out of 5), increases in knowledge (4.2/5) and confidence (3.9/5) towards managing stress, increases in knowledge (4.1/5) and confidence (3.9/5) in showing support and care towards family members, and an increase in knowledge towards strengthening family relationships (4.0/5). Phase two participants (*n* = 294) also reported high satisfaction (4.7/5) and strongly agreed that ICT helped with learning Zentangle more conveniently, that they had increased knowledge and interest in Zentangle (all 4.7/5), and would definitely join the workshop again (4.8/5). The qualitative data supported the quantitative findings. We are the first to report on the utilization of ICT in an exploratory trial of brief, online Zentangle art workshops targeting the general public, with high satisfaction and positive participant experiences with ICT integration, learning Zentangle, and enhanced psychological and family well-being. This study provided preliminary evidence on the use of ICT to successfully transition face-to-face to online workshops and reach a wider audience.

## 1. Introduction

The Zentangle^®^ method is a mindfulness-based art therapy in which participants create detailed images through drawing repetitive structured patterns, called *tangles*, with intricate dots, lines, and curves [1]. These abstract patterns consist of just one to three simple strokes drawn on small pieces of paper called *tiles*, which can be assembled into large mosaics. Created by Rick Roberts and Maria Thomas in 2003, Zentangle is unique in that it requires little prior drawing skills, planning, or equipment to start. Patterns are drawn freely, tiles can be rotated in any direction, and no corrections are involved. It is easy to learn and suitable for anyone to do anywhere, even in the comfort of their own homes.

The simplicity and meditative nature of Zentangle art has propelled its popularity in recent years [2,3,4], including in Hong Kong [5]. The current global Zentangle community is wide-reaching, with over 3000 certified teachers in over 40 countries [1]. However, our search of *PubMed*, *Scopus*, and *Education Resources Information Center* (ERIC), an education literature and resources database, using the keyword “Zentangle” on 1 April 2022 yielded only two studies. One reported on the integration of Zentangle into a children’s education program to enhance motor and language skills and self-confidence [6] but conducted evaluation mainly through observation of only forty children in two kindergarten classes. The other reported a Zentangle art intervention on rural healthcare workers to reduce workplace stress [7] but was limited by a small sample size of 40 participants. We found no reports of intervention studies on Zentangle, online or offline, targeted at the general public.

Mindfulness is most commonly defined as a present-focused awareness that is non-reactive and non-judgemental [8,9]. Mindfulness-based interventions are effective in improving various psychosocial conditions, including depression, anxiety, insomnia, hypertension, and pain [10], and have shown to reduce distress, promote emotional regulation, and enhance psychological health and holistic well-being [9,11,12]. Art therapy is a therapeutic intervention that utilizes and integrates the creative process of art making to improve physical, mental, and emotional well-being [13]. Mindfulness-based art therapy (MBAT), specifically, has complementary effects on mental health [14] and has been shown to induce emotional relaxation; reduce stress, depression and anxiety symptoms; and enhance spiritual well-being and quality of life [15,16,17,18,19,20]. However, these interventions are usually resource- and time-intensive, and take several months to a year before effects can be detected. Many MBAT interventions are also targeted towards patients instead of the general population [15,19,20,21].

Zentangle avoids such limitations with its simplicity and creativity through imagination and relaxation that is attractive for all age groups. It allows for individuals who do not feel artistic to be creative and innovative and can be taught quickly with just one short session (i.e., a 30 min workshop). The physical, tangible end product created can be a symbol of achievement and even be shared with or gifted to others.

While Zentangle does emphasize mindfulness, it mainly focuses on promoting gratitude and appreciation through focused drawings of the structured patterns [1]. Pens are preferred over pencils to encourage acceptance and reframing, and erasers are not needed, as the process does not recognize mistakes, which should instead be reframed as opportunities. Participants can simply enjoy the present moment and the mindful and relaxing process of creating Zentangle art. Once participants are taught the basic techniques and patterns, they can create art anywhere and at any time, with just a pen and a piece of paper.

The Hong Kong Jockey Club SMART Family-Link (JCSFL) Project, launched in 2018, is a four-year, interdisciplinary, cross-sectoral collaboration between academia (School of Public Health (SPH) and Technology-Enriched Learning Initiative (TELI) of The University of Hong Kong (HKU)) and family social services (26 Integrated Family Service Centres (IFSCs) operated by 12 non-governmental organizations (NGOs)). The aim is to promote family well-being in the community through advancing the use of information communication technologies (ICT) [22]. One of the five main components of the JCSFL Project is i-Action, which involves the co-creation of innovative and preventive interventions leveraging ICT to enhance family functioning and family service delivery [23], and deep collaboration with and support for IFSCs in their preventive family services.

Caritas—Hong Kong, one of the JCSFL Project’s collaborating partners, is a charitable NGO offering a wide range of services to different sectors of the community. Caritas Family Service offers assistance to individuals and families through a variety of programs, with the aim to preserve and strengthen families by improving their quality of life and ability to deal with problems within the family unit [24].

Caritas Integrated Family Service Centre—Aberdeen (Caritas Aberdeen IFSC) began independently conducting face-to-face Zentangle workshops in 2018 as a new and easy approach to promote effective stress management and reduce negative psychological feelings in their service users. Amidst the social unrest in the city that began in June 2019 [25], workshops specifically aimed for individuals and couples experiencing related stress were conducted.

In collaboration with the JCSFL Project, Caritas Aberdeen IFSC conducted the first Zentangle workshop under i-Action in September 2019 as part of a four-session, face-to-face workshop to promote stress management and family well-being. With the first confirmed case of the novel coronavirus disease (COVID-19) in Hong Kong in late January 2020 [26], Caritas Aberdeen IFSC quickly transitioned from traditional face-to-face service provisions to online by utilizing ICT and began hosting online, single-session Zentangle workshops in February 2020 via Facebook Live. With the emergence of the pandemic compounded with months of social unrest in the city, simple and brief interventions to help enhance well-being were urgently needed [27,28].

We reported the transition from face-to-face to online Zentangle workshops utilizing ICT in family social services during COVID-19. We explored feedback from face-to-face Zentangle workshops and the acceptability of an online approach to achieve greater service reach, satisfaction, and knowledge and related outcomes, with deep cross-sectoral collaboration between HKU and Caritas Aberdeen IFSC under the JCSFL Project.

## 2. Materials and Methods

### 2.1. Study Design

**Figure 1:**
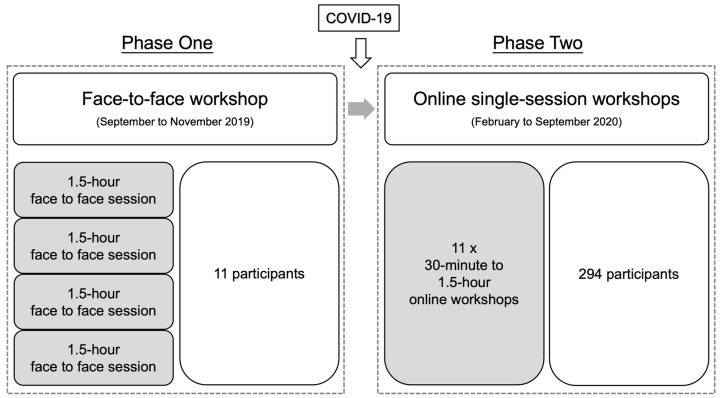
Study phases and design of the Caritas Aberdeen Integrated Family Service Centre (IFSC) Zentangle art program.

This study was conducted in two phases: a Zentangle service program comprised of a face-to-face workshop (phase one) and online single-session workshops (phase two) from September 2019 to September 2020 (Figure 1). Each Zentangle workshop was led by a certified Zentangle teacher and experienced social worker (EN) from Caritas Aberdeen IFSC. Registration was free, open to the public, and on a first-come-first-served basis. Recruitment and promotion were primarily conducted via the Caritas Aberdeen IFSC Facebook page and posters placed in the centre. Eligibility criteria included: (i) had access to the internet; and (ii) able to complete a simple questionnaire. Caritas Aberdeen IFSC social workers obtained informed written consent from all participants. As this Zentangle program aimed to reach 300 annual participants, COVID-19 prompted the online transition to maintain service delivery.

#### 2.1.1. Phase One—Face-to-Face Workshop

A four-session (1.5 h each) Zentangle workshop was conducted during 27 September to 1 November 2019 at the Caritas Aberdeen IFSC for individuals or couples who experienced stress due to the social unrest in the city that began in June 2019, with the aims of enhancing stress management and family well-being. Limited by the size of the classroom, 11 participants were recruited, and they attended all four sessions.

#### 2.1.2. Phase Two—Online Single-Session Workshops

A total of eleven online Zentangle workshops (ranging from 30 min to 1.5 h) were conducted via Facebook Live hosted on the Caritas Aberdeen IFSC Facebook page (https://www.facebook.com/caritas.abnifsc/, accessed on 2 July 2021) [29] during February 2020 to September 2020. In response to the pandemic, Caritas Aberdeen IFSC began hosting free, online single-session workshops with the aims of reaching as many people as possible amid social distancing measures and providing an avenue of support to service users. Although the online format allowed for many more participants, registration was still mandatory to ensure their eligibility. The majority of participants joined the workshops via computers and smartphones.

### 2.2. Interventions

**Figure A1:**
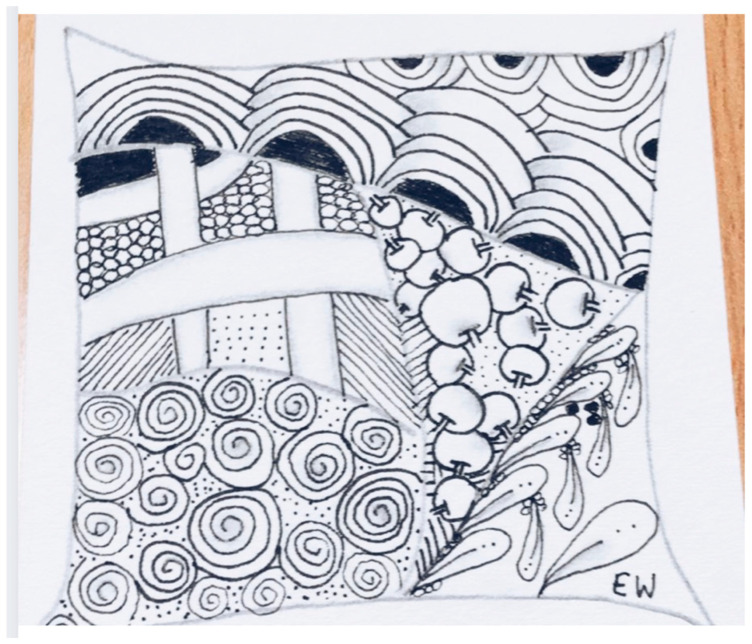
Sample Zentangle artwork created in one workshop (five patterns, left bottom to top right: printemps, hollibaugh, pokeroot, flux, and crescent moon).

Workshop content and implementation in both phases were similar, differing only in delivery mode and duration. Each workshop comprised three sections: introduction, demonstration and drawings, and summary and sharing (Table 1), with a participant’s sample artwork shown in Figure A1. At the end of each session of the face-to-face workshop, participants would combine their tiles together into a large mosaic for a group photo. Alternatively, online participants shared their artwork with Caritas Aberdeen IFSC either via WhatsApp (prior to July 2020) or the Facebook group, 「禪圈」 or “Zen Ring” (www.facebook.com/groups/ring.zen/, accessed on 2 July 2021), created by Caritas Aberdeen IFSC in July 2020 and aptly named to symbolize a circle of friends who collectively enjoy Zentangle.

**Figure A2:**
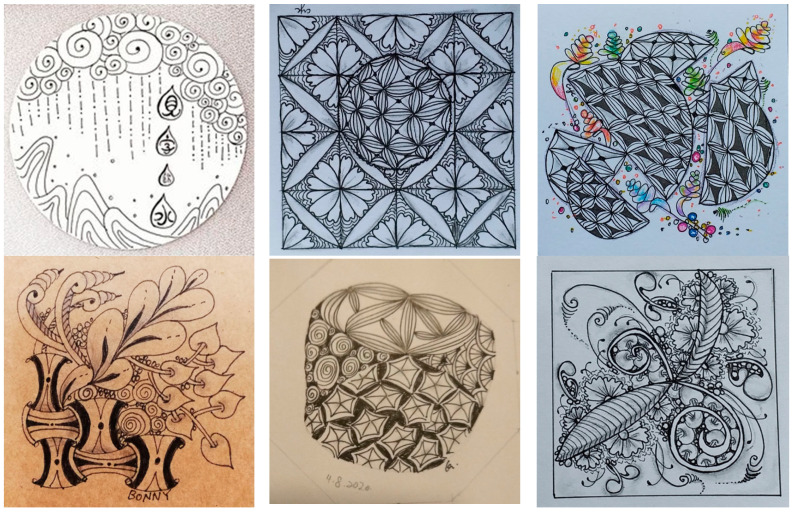

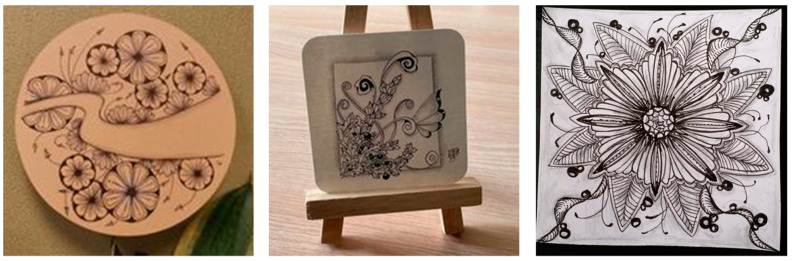
Participants’ Zentangle artwork posted on Caritas Aberdeen IFSC’s Facebook or the “Zen Ring” Facebook Group during September 2019 to September 2020.

**Figure A3:**
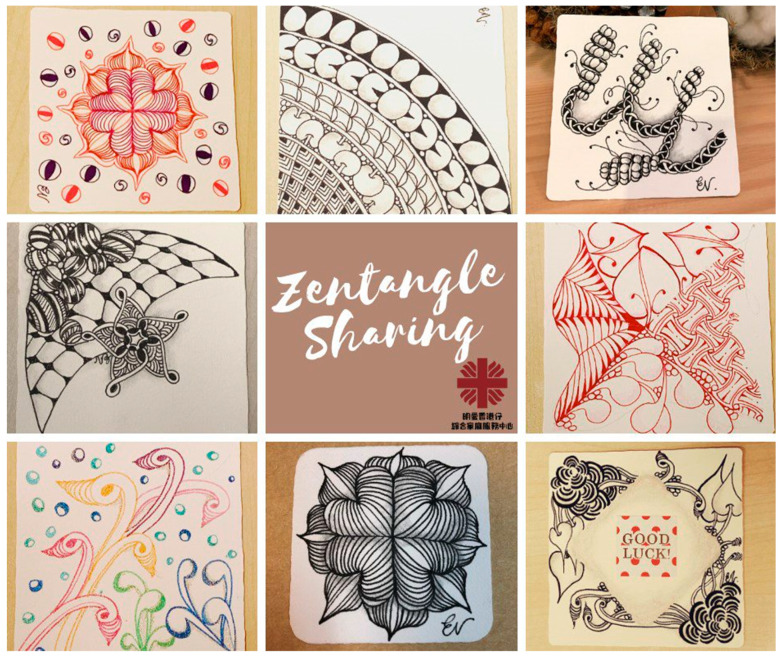

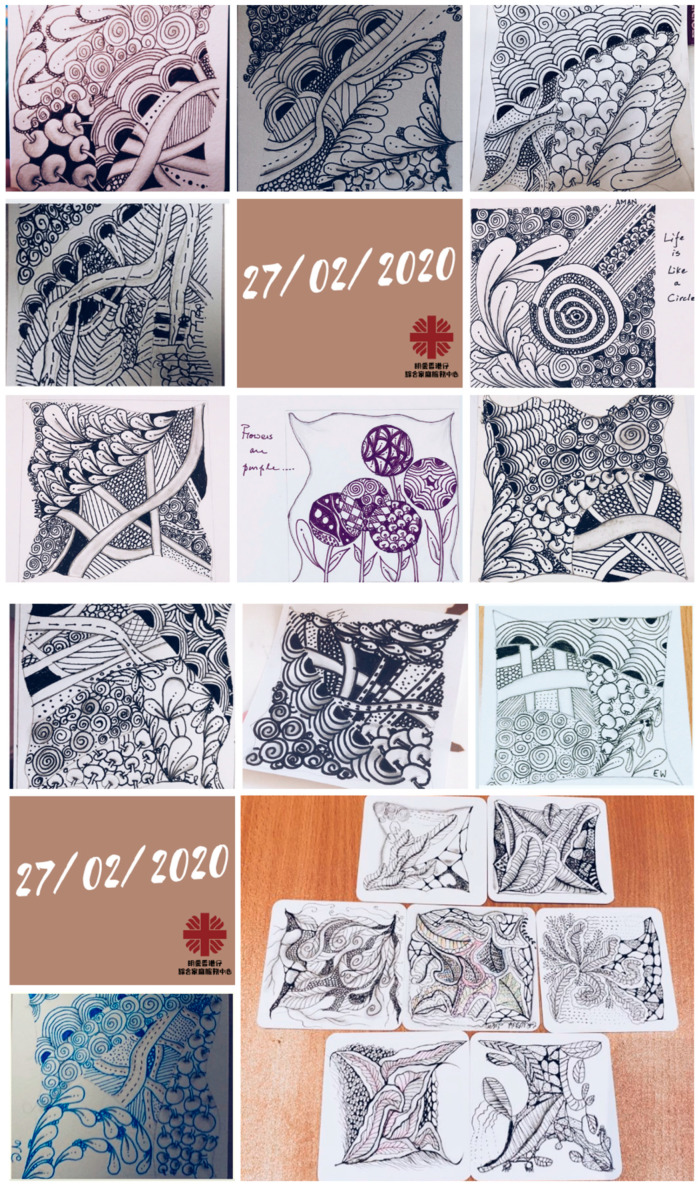
Collages of participants’ Zentangle artwork from different workshops posted by Caritas Aberdeen IFSC on their Facebook during September 2019 to September 2020.

There are currently more than one hundred pattern variations of official Zentangle tangles, each with a different name and technique (such as *hollibaugh*, consisting of a series of two parallel lines reminiscent of roads, and *printemps*, small spirals reminiscent of jelly rolls). Examples of participants’ created Zentangle art and workshop collages are shown in Figure A2 and Figure A3. The workshops also incorporated the additional use of 「智放鬆」 or “SMART Relaxation”, a game application designed for mindfulness with different tracks of calming background music accompanied by beautiful landscape wallpapers, and a built-in feature that allows users to set the length of time of music played without interruptions. This was one of over a dozen free game applications developed under the Project to promote family-related themes such as coping and communication (https://jcsmartfamilylink.hk/appindex/, accessed on 2 August 2021).

### 2.3. Community Promotion and Sharing

After receiving overwhelmingly positive feedback from service users, all with deep appreciation and interest in Zentangle, Caritas Aberdeen IFSC created the “Zen Ring” Facebook group on 3 July 2020, dedicated to the sharing and mutual appreciation of Zentangle art. It allows group members and the general public to post their own art and receive support and appreciation from others, to share their experiences and takeaways from the workshops, and to encourage everyone to create art in their own time. It also serves as an interactive platform for Caritas Aberdeen IFSC to disseminate and others to access more information on Zentangle. The group fosters a wide community network of mutual support and sharing of Zentangle art across Hong Kong and beyond. It facilitates further interactions and connections between community participants and Caritas Aberdeen IFSC social workers.

All online workshops hosted via Facebook Live were recorded and made publicly available on the Caritas Aberdeen IFSC Facebook page for further viewing and sharing. Participants and community members can access past workshops at any time to learn and review lessons and techniques, with Facebook links of all 11 workshops (including detailed introduction and step by step guides) provided in Table A1.

### 2.4. Data Collection

For the face-to-face workshop, a questionnaire survey was administered immediately after the fourth (and last) session. Questions on participant satisfaction, reactions to the program, stress management, and family well-being were asked. The sex and approximate age groups of participants were recorded through observation by Caritas Aberdeen IFSC social workers.

Immediately after each online workshop, participants completed an online questionnaire via a QR code displayed on the screen, and a corresponding web link was also provided via the chat box. Questions included both process and outcome evaluation items and an open-ended question for qualitative feedback or suggestions related to the workshop. As this was an exploratory trial, evaluation was kept simple and brief, follow-up surveys were not administered, and demographic information was not collected. However, Facebook data were available to provide simple, supplementary demographic information, specifically the main demographic group (sex and age group) that joined each Facebook Live workshop. Data on the number reached (number of times each online workshop was displayed on a unique screen), the number of views (number of times each online workshop was watched) and interactions (number of likes, comments, shares, or reactions each online workshop received) were also collated. Participants also shared their Zentangle art after the workshops via the Caritas Aberdeen IFSC Facebook page and “Zen Ring” Facebook group.

Additionally, as part of a promotional campaign for the JCSFL Project in promoting the importance of family well-being, one of the couples that attended the online Zentangle workshops were invited to partake in a taped video interview on their experiences with Zentangle art and the workshops and the impact of learning Zentangle on their psychological and family well-being. The interview was written as an article in the “Striding On” magazine, a quarterly publication by the Hong Kong Jockey Club Charities Trust, to highlight important stories and achievements in promoting well-being in Hong Kong [30]. Qualitative data were extracted from this interview based on the transcript, which was analysed using thematic framework analysis [31].

### 2.5. Measures

#### 2.5.1. Phase One

##### Process Evaluation

All participants were asked to rate their overall satisfaction towards the workshop with the question, “Overall, how satisfied are you with this workshop?”, with responses on a 5-point Likert scale, ranging from 1 (very unsatisfied) to 5 (very satisfied).

Applicability of the workshop content in everyday life was assessed by the question, “Is the content from this workshop applicable in everyday life?” with similar responses from 1 (not at all) to 5 (very much).

Sharing and recommendation of the workshop to others was assessed by two questions: “Will you share the contents and happy things from the workshop with family/friends?”, and “Would you recommend this workshop to others?”, with response options of “Yes” or “No”.

##### Outcome Evaluation

Knowledge and confidence towards managing stress were assessed by two questions: “Compared to before joining this workshop, has your knowledge/confidence in managing stress increased?”.

Knowledge in strengthening family relationships was assessed by the question, “Compared to before joining this workshop, has your knowledge in strengthening family relationships increased?”.

Knowledge and confidence in showing care and support towards family members were assessed by two questions: “Compared to before joining this workshop, has your knowledge/confidence in showing support and care towards family members increased?”. All the above questions had responses from 1 (not at all) to 5 (very much).

#### 2.5.2. Phase Two

##### Process Evaluation

In addition to one question on overall satisfaction towards the workshop, participants were asked to rate the integration of ICT into the workshop by the question, “Did ICT help with learning Zentangle more conveniently?” with responses from 1 (not at all) to 5 (very much).

The desire to join the Zentangle workshop again was assessed by the question, “Would you join this workshop again?” with responses from 1 (no) to 5 (definitely).

##### Outcome Evaluation

Knowledge and interests in Zentangle were assessed by two questions: “Did the workshop increase your knowledge of/interests in Zentangle?”, with responses from 1 (not at all) to 5 (very much).

## 3. Results

A total of 305 participants joined Caritas’ Zentangle face-to-face workshop (11 participants) or online single sessions (294 participants) during September 2019 to September 2020.

### 3.1. Phase One—Face-to-Face Workshop

#### 3.1.1. Process Evaluation

Table 2 shows the 11 participants (91% female, all adults aged around 40 or above) reported high satisfaction towards the Zentangle workshop (4.7 ± 0.65) and applicability of workshop contents in everyday life (4.3 ± 0.65). Almost all participants reported they would share content and happy things from the workshop with family (91%) and friends (100%). All participants reported they would recommend this workshop to others.

#### 3.1.2. Outcome Evaluation

Participants reported an increase in knowledge (4.2 ± 0.63) and confidence (3.9 ± 0.57) towards managing stress and an increase in knowledge (4.1 ± 0.54) and confidence (3.9 ± 0.54) in showing support and care towards family members. They also reported an increase in knowledge towards strengthening family relationships (4.0 ± 0.67).

### 3.2. Phase Two—Online Single Sessions

Of the 294 participants that joined the 11 online workshops (average of 27 participants per workshop), Facebook data identified females (ranging from 74% to 91%) and individuals aged 35 to 44 years as the main demographic groups for all workshops.

#### 3.2.1. Process Evaluation

**Figure 2:**
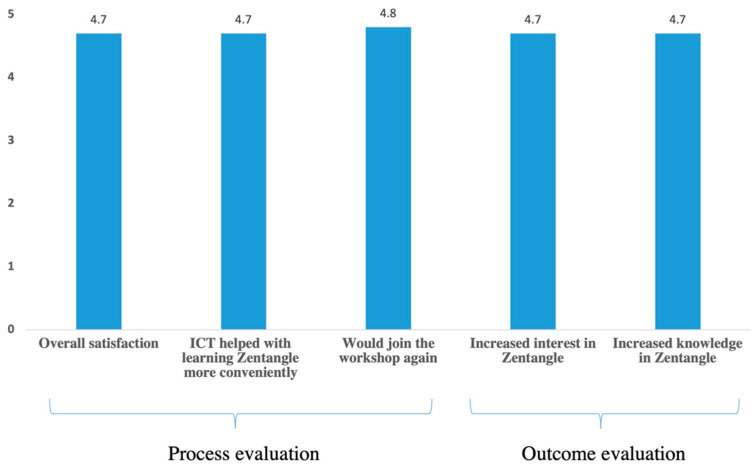
Overall satisfaction and perceived changes in knowledge and attitudes of Caritas Aberdeen IFSC’s online Zentangle workshops (Phase 2, *n* = 294).

Figure 2 shows participants reported a high overall satisfaction score, strongly agreed that ICT helped with learning Zentangle more conveniently (both with mean scores of 4.7 out of 5), and said that they would definitely join the workshop again (mean 4.8 out of 5).

Participant feedback provided in the online questionnaire and video-recorded interview was highly positive and consistent with these findings, with some of their words quoted below:

Feedback from workshop participants (submitted anonymously):

*“The teacher is so great and patient, I’m very grateful.”*

*“Very enjoyable class, the teacher is so clear in explaining everything.”*

*“We don’t need to leave home to go to class, and can even save on transportation time… Please host more online workshops so we can join and learn conveniently from home!”*

*“I really appreciate the teacher’s patience in teaching, but due to the online format, sometimes her hand blocked the camera during the demonstrations.”*

*“This class was great, please continue to conduct workshops online, as it has allowed me to learn while taking care of my children… there have been very few online courses in the past, so it was difficult to participate.”*

Feedback from the video-recorded interview (female, aged around 60):

*“Because of COVID-19, the online learning format is great, as we can keep learning from our instructor virtually even with social distancing …”*

*“Learning Zentangle face-to-face has its advantages… sitting together in small group where you can chat together while drawing is a great feeling… Interaction is always best. But given the current situation, the online format is a good alternative method… and allows you to revisit previous class content.”*

#### 3.2.2. Outcome Evaluation

Figure 2 also shows the workshops greatly increased participants’ knowledge and interests in Zentangle (both with mean scores of 4.7 out of 5). Almost all participants reported they would share the workshop contents and happy things with family (91%) and friends (100%), and all would recommend the workshop to others (100%).

Some of the participants’ feedback is quoted below:

*“Very good class… Zentangle is simple and easy to learn.”*

*“This was a great introduction into Zentangle… I want to learn more advanced techniques.”*

*“I’m really hoping there are more Zentangle classes in the future.”*

*“The teacher’s art is so beautiful, hoping to learn more of her techniques in the future.”*

#### 3.2.3. Stress Reduction and Enhancement of Psychological and Family Well-Being

Many participants shared that the workshops made them feel more relaxed and less stressed and facilitated increased interactions with their families. Some of their words are quoted below:

Feedback from workshop participants:

*“Learning Zentangle through Facebook Live makes me feel relaxed… I can learn with no stress or pressure.”*

*“I feel no stress through this online class… I can also revisit the class content.”*

*“I can do this together with the whole family… it’s a very good parent–child and family activity.”*

Feedback from the video-recorded interview:

*“Makes me very happy to receive this art from my husband. I give him 10 out of 10, full marks!”*

*“I helped my son and husband sign up for Zentangle as well because not only did I personally enjoy the class, I wanted my family to experience it together so that we have a common topic for discussion…”*

*“The drawing process is also very relaxing and calming. As my son has quite a stressful and challenging curriculum, I hoped that joining this activity could help relieve some of his stress and I think it has…”*

#### 3.2.4. Suggestions for Future Zentangle Programs

Participants also provided suggestions for future program implementation, including wanting additional online classes and workshops catered to individuals of different skill levels:

*“A set class schedule with at least one workshop every one or two weeks would be best. Please host more workshops!”*

*“Please spend more time on teaching the techniques, like shading and drawing smooth, long lines. I want to get better.”*

*“I think the workshops would work best if the participants already had some prior experience or basic drawing skills to be able to follow the instructor more easily.”*

*“Might be because I’m a beginner, but I felt that I couldn’t keep up with the teacher at times.”*

*“More interactions during the online class would be nice.”*

### 3.3. Continuous Community Promotion

**Figure 3:**
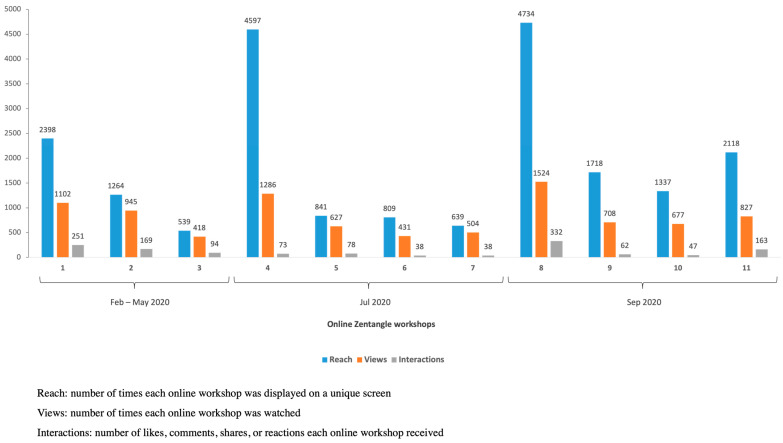
Overview of Facebook Live reach, views, and interactions of 11 online Zentangle workshops.

**Figure 4:**
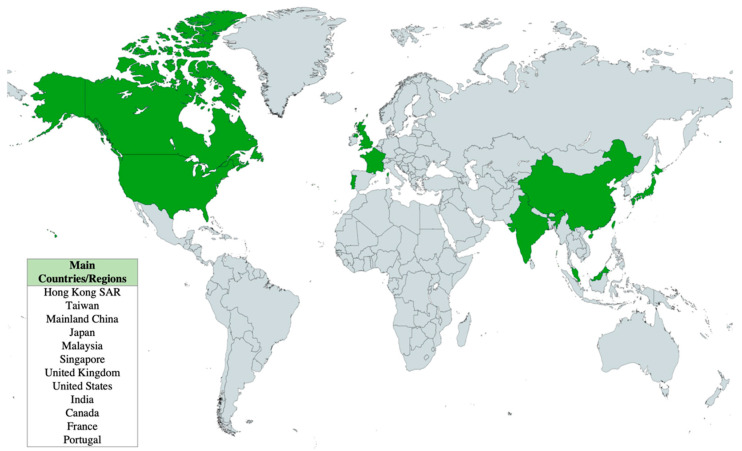
Locations of Caritas Aberdeen IFSC’s online Zentangle workshop viewers.

Facebook data showed high reach, views, and interactions across all online workshops. As of 24 April 2022, the workshops accumulated a total reach of 20,994, with 9049 views and 1345 interactions (Figure 3). Patterns taught in the top three viewed workshops were: crescent moon, flux, hollibaugh, pokeroot, printemps (workshops #1 and #8), and drupe and fracas (workshop #4). Viewers of the workshops were based in 12 countries or regions, including the United States, United Kingdom, France, and Portugal (Figure 4).

As of the same date, the “Zen Ring” Facebook group had a total of 942 members with frequent (almost daily) sharing and uploads of Zentangle art from community participants [29]. Over the past year (24 April 2021 to 23 April 2022), the group had a total of 39,626 visits, 526 posts (average 1.4 posts per day), 9283 reactions, and 539 comments. As Facebook only allows access to data for the past year, data from the start date on 3 July 2020 until 23 April 2021 were not included, and so these numbers should be even greater.

**Figure 5:**
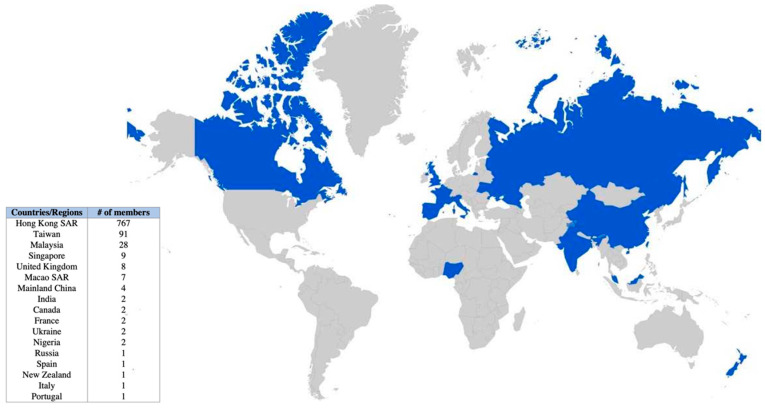
Locations of Caritas Aberdeen IFSC’s “Zen Ring” Facebook group members.

Among the members that joined the “Zen Ring” group in the past year, the majority were female (86%) and aged over 35 (50% from 35 to 54, 25% over 55). Figure 5 shows the extensive global reach of the group, with members from 17 countries or regions, with most members from Hong Kong SAR, Taiwan, Malaysia, and Singapore.

## 4. Discussion

This is the first report on the utilization of ICT in an exploratory trial of brief, online Zentangle art workshops targeting the general public, with high participant satisfaction towards the overall program and online format and positive experiences with ICT integration and learning Zentangle. Participants also reported enhanced psychological and family well-being. Almost all participants reported they would share the workshop contents and recommend the workshop to their family and friends. Facebook data showed high reach, views, and interactions across all online workshops and the global reach of the online Zentangle group. The qualitative data further supported and enriched the findings.

Our online Zentangle art intervention differs from other art therapy interventions reported in the literature [32,33], as Zentangle is easy to learn with few barriers and does not need special skills and equipment, preparation, or money and resources. Individuals of all ages and abilities can draw anywhere, at any time, with just a pen and paper. In fact, a previous Zentangle intervention was successfully conducted in kindergarten classrooms with children aged 3 to 5 years to enhance their fine motor skills, language experience, and self-confidence [6]. Our intervention can also be easily implemented, as it is brief (30 min), inexpensive, and should be cost-effective to disseminate.

Participants of both phases reported enhanced psychological and family well-being, which has not been found in previous peer-reviewed journal papers. As Zentangle focuses on mindfulness, gratitude, and appreciation, this type of mindfulness-based art therapy allows participants to enter a meditative state while drawing. Art therapy practice involving engagement in structured designs such as Zentangle can enhance focused awareness and develop mindfulness [11], which boost self-esteem and self-regulation, and promote cognitive function and psychological and physical health [34]. Engaging in repetitive and creative behaviours, such as drawing lines and patterns, can be very calming and help neutralize anxiety [3]. Almost all participants reported they would share the contents from the workshop and happy things with their families, and their qualitative feedback indicated Zentangle art can help foster more family communication and interactions.

Under the JCSFL Project’s i-Action, the utilization of ICT facilitated Caritas Aberdeen IFSC to successfully promote and conduct a small, traditionally face-to-face family service program online. Our social workers were also able to reach and benefit a wider network of people in the community far away from the centre and extend our reach beyond Hong Kong to distant parts of the world. Our findings highlight the potential in such an approach to maximize service reach with minimal resources and costs. The online format was also well-accepted by participants, as many agreed that it allowed them to learn Zentangle more conveniently and that they would like to join more future online workshops. With the use of ICT, Caritas Aberdeen IFSC was also able to foster a supportive community network through the Caritas “Zen Ring” Facebook group, which continues to expand, currently reaching a global audience from over 17 countries and regions.

Uncertainties of the pandemic and social distancing measures have caused fear, loneliness, poor emotional well-being, and increased family conflicts [27,35,36]. Many essential services have also faced significant challenges, including those of the social service sector [37]. Amidst such circumstances, Caritas Aberdeen IFSC recognized the need for an alternative solution to assist their service users, and their proactivity and creativity allowed for the successful implementation of online Zentangle workshops that benefitted many members of the community in Hong Kong and around the world. In collaboration with the JCSFL Project, positive results and experiences stemming from this exploratory trial have provided preliminary evidence on the use of ICT for implementing online programs, and will guide the development of future Zentangle programs by other IFSCs of Caritas as well as other i-Action and family service programs in collaboration with IFSCs of other NGOs. Our results also indicate the further potential and feasibility of additional brief, online interventions to meet the growing demand for mental health support and achieve greater reach and penetration in the community. However, whether individuals with negative emotions and poor psychological well-being would be benefitted needs to be explored as they are less reachable.

This study had a few limitations. As this was an exploratory trial to assess satisfaction and applicability of an online program, the evaluation questionnaire was kept very short, and simple demographic information was only collected through Facebook. This was not a randomised controlled trial and did not have a control group and no follow-up for longer-term outcomes. Future studies, particularly randomised controlled trials, with more in-depth evaluation and follow-up and use of validated questionnaires to examine psychological and family well-being are needed. The workshops were also quite short (30 min), so future programs can explore whether classes with a longer duration or more frequent sessions (as suggested by participants) that increase the intensity of the intervention can increase participant satisfaction and yield longer-term impacts. Our sample was also based on participants who were interested and registered for the workshops and had access to either the face-to-face or virtual format. This self-selection method sampled only a subset of the population, which may have resulted in volunteer bias; thus, the findings should be interpreted with caution.

## 5. Conclusions

Our Zentangle service program with face-to-face and online workshops showed high satisfaction and positive participant experiences with ICT integration, learning Zentangle, and enhancing psychological and family well-being. Participants reported high interests in joining future online workshops. With COVID-19 causing disruptions to mental health service capacity and delivery, especially for family social services, this study provided preliminary evidence on the use of ICT to successfully transition face-to-face to online programs and reach a wider audience, which can help the development of future Zentangle and family service programs. Our results also indicate further potential and feasibility of additional brief online interventions to meet the growing demand for mental health support and achieve greater service reach and penetration.

## Figures and Tables

**Table 1:** Overview and structure of a Zentangle workshop.

Section	Topic	Activity ^a^
1	1. Introduction 2. Workshop purpose and goals	- Introduction of the Zentangle method and brief history - Guide participants to set intentions of mindfulness, gratitude, and appreciation for the workshop - Overview of patterns and techniques that will be taught
2	Demonstration and drawing	- Step by step explanation and demonstration of each pattern while guiding participants to create their own unique art, including direction and placement of strokes, pencil pressure, and shading - Calming music played in the background to encourage relaxation in the process and promote creativity Example of 5 patterns taught in one workshop (with sample artwork shown in Figure A1): printemps, hollibaugh, pokeroot, flux, and crescent moon
3	Summary and sharing	- Interactive sharing among participants on the created artworks and their positive experiences and challenges from the workshop - Group photo taking

^a^ For the face-to-face workshop, different patterns were taught in each of the four sessions.

**Table 2:** Evaluation of Caritas Aberdeen IFSC’s face-to-face Zentangle workshop (Phase 1, *n* = 11).

Phase 1 (*n* = 11) *	Average Score (±SD)	Satisfaction Score of 4 or 5 (%)
**Process evaluation**		
Reactions to the intervention content and design		
Overall satisfaction ^a^	4.7 ± 0.65	91
Applicability of workshop content in everyday life ^b^	4.3 ± 0.65	91
	*Answered “Yes” (%)*	
Sharing and recommendation of workshop ^c^		
Would share content and happy things from workshop with family	91	
Would share content and happy things from workshop with friends	100	
Would recommend this workshop to others	100	
**Outcome evaluation**		
Stress management ^d^		
Increase in knowledge towards managing stress	4.2 ± 0.63	90 ^^^
Increase in confidence towards managing stress	3.9 ± 0.57	80 ^^^
Family well-being ^d^		
Increase knowledge towards strengthening family relationships	4.0 ± 0.67	80 ^^^
Increase knowledge in showing support and care towards family members	4.1 ± 0.54	91
Increase confidence to show support and care towards family members	3.9 ± 0.54	82

^a^ Scale of 1 (very unsatisfied) to 5 (very satisfied); ^b^ Scale of 1 (very unapplicable) to 5 (very applicable); ^c^ Yes or No answer options; ^d^ Scale of 1 (not at all) to 5 (very much); ^^^ *n* = 10; * Participants included 10 females and 1 male.

## Data Availability

The data presented in this study are available on request from the corresponding authors.

## Appendix A

**Table A1:** Facebook links to all Caritas Aberdeen IFSC online Zentangle workshops during February to September 2020.

Workshop	Facebook Link
1	https://www.facebook.com/caritas.abnifsc/videos/192005762024507/
2	https://www.facebook.com/caritas.abnifsc/videos/856816548062996/
3	https://www.facebook.com/caritas.abnifsc/videos/1178499732482937/
4	https://www.facebook.com/caritas.abnifsc/videos/3072814369420540/
5	https://www.facebook.com/caritas.abnifsc/videos/3348053791927255/
6	https://www.facebook.com/caritas.abnifsc/videos/324510075259141/
7	https://www.facebook.com/caritas.abnifsc/videos/324510075259141/
8	https://www.facebook.com/caritas.abnifsc/videos/715310032397086/
9	https://www.facebook.com/caritas.abnifsc/videos/749816099141491/
10	https://www.facebook.com/caritas.abnifsc/videos/1628872087295091/
11	https://www.facebook.com/caritas.abnifsc/videos/344190326696217/

Accessed on 2 August 2021.
